# Exploring Clinical Trajectories and the Continuum of Care for Patients With Acute Coronary Syndrome in the United Kingdom: A Thorough Cross-Sectional Analysis

**DOI:** 10.7759/cureus.49391

**Published:** 2023-11-25

**Authors:** Hany A Zaki, Israr Bashir, Ahmed Mahdy, Mohammed Abdurabu, Hosam Khallafalla, Mohamed Fayed, Wael Abdelrehem Elnabawy Elsayed, Mohammed Gafar Abdelrahim, Kaleem Basharat, Wathek Salloum, Eman Shaban

**Affiliations:** 1 Emergency Medicine, Hamad Medical Corporation, Doha, QAT; 2 Cardiology, Al Jufairi Diagnosis and Treatment, Doha, QAT

**Keywords:** rehabilitation, hospitalization, heart, emergency care, cardiovascular disease

## Abstract

The United Kingdom (UK) has a sustainable healthcare system. Nonetheless, the burden of acute coronary syndrome (ACS) is still a significant challenge. A scarcity of literature primarily focuses on the continuum of care for ACS patients in the UK. Moreover, limited research studies highlight the clinical trajectories of ACS patients across the UK. Therefore, the current study was designed to explore clinical trajectories and the continuum of care for patients with ACS in the UK.

Secondary data was obtained from the Myocardial Ischaemia National Audit Project (MINAP) database. The latest data available in the MINAP database was used. As our objective was to explore clinical trajectories and the continuum of care for patients, we retrieved data regarding the care received by ACS patients admitted to hospitals across the UK.

The data of 85574 ACS patients was retrieved. A large number (n=47035) of patients were estimated to be eligible for the angiogram; however, an angiogram was performed for 87.15% (n=40995) of eligible patients. Angioplasty within 72 hours of admission was required for most (n=26313) ACS patients. Nonetheless, angioplasty within 72 hours of admission was performed for 59.7% (n=15703) of the eligible patients. There was a significant difference (P<0.05) between different regions of the UK and the percentage of patients for whom angioplasty was performed within 72 hours of admission. Primary percutaneous coronary intervention (PCI) was performed for 23923 ACS patients, of which the door-to-balloon interval for 17590 (73.5%) patients was ≤60 minutes while the door-to-balloon interval for 3086 (12.9%) patients was ≤90 minutes. Out of the total 85574 ACS patients, 65959 (77.08%) patients were discharged on appropriate medications, while 19615 (22.92%) were transferred to another hospital or died there. A total of 75361 were eligible to be referred to cardiac rehabilitation settings. Nonetheless, 64518 (85.61%) were referred to cardiac rehabilitation.

About 85000 patients were reported in the UK (England, Northern Ireland, Wales). Optimal care was provided to most patients in the UK. However, some patients received sub-optimal care, highlighting the disparity in the healthcare system. There is a need to explore further the factors that might be responsible for the sub-optimal care to the patients.

## Introduction

Cardiovascular diseases (CVDs) are the leading causes of death throughout the world, and according to the World Health Organization (WHO), about 17.9 million people die annually due to CVDs [1-3]. Of CVDs, acute coronary syndrome (ACS) is the most significant single cause of death, as almost half of CVD-related deaths are attributable to ACS [4]. Similarly, ACS is responsible for 12% of disability-adjusted life-years lost globally [5]. Though ACS epidemiology varies between high-income and low and middle-income countries (LMICs), the literature suggests that the burden of ACS and its economic consequences are substantial in high-income countries and LMICs [6,7].

The United Kingdom (UK), a high-income country, has a sustainable healthcare system. Nonetheless, the burden of ACS is still a significant challenge [8-10]. About 2.3 million UK citizens (1.5 million male and 0.8 million female) suffer from ACS, and about 66000 people in the UK die due to ACS each year [11-13]. ACS is the most common cause of premature mortality in the UK. Annually, about 25000 individuals with age less than 75 years old die due to ACS [14,15]. The annual cost of CVDs in the UK is estimated to be £9 billion, and ACS is responsible for about 75% of this economic cost [14,16].

Evidence suggests that ACS patients receiving emergency services and care during hospitalization and rehabilitation stages significantly affect ACS outcomes [17,18]. The fact is that the management of ACS during pre-hospitalization, hospitalization, and post-hospitalization is equally important as the short- and long-term outcomes of ACS depend on the attention patients receive during all these three stages [19,20]. Previous research studies conducted in the UK either focused on specific intervention strategies used for treating ACS or on the care level received by ACS patients at particular stages of the disease [21-23]. A scarcity of literature primarily focuses on the continuum of care for ACS patients in the UK. Moreover, limited research studies highlight the clinical trajectories of ACS patients across the UK. Therefore, the current study was designed to explore clinical trajectories and the continuum of care for patients with ACS in the UK.

## Materials and methods

Secondary data was obtained from the Myocardial Ischaemia National Audit Project (MINAP) database (NICOR | Myocardial Ischaemia/MINAP (Heart Attack audit)). The MINAP database contains data regarding the care received by ACS patients presented to hospitals across the UK. The data present in the MINAP database is collected from hospitals in England, Northern Ireland, and Wales. Data related to various domains of patient care are updated at regular intervals. At present, MINAP is the single largest ACS registry in the UK and contributes to the National Cardiac Audit Programme (NCAP) of the UK. Through the audit process, the MINAP initiative seeks to assess and enhance the clinical care and, consequently, the outcomes of patients with ACS.

Additionally, it aims to supply valuable high-resolution data for research. MINAP has established a minimum standard for data completeness to maintain the data quality. MINAP holds data of only those cases for which at least 95% of data is available [24]. 

The MINAP database was established in 1998 and is updated annually. The latest data available in the MINAP database was for 2021-22; for the current study, we utilized 2021-22 datasets. Our objective was to explore clinical trajectories and the continuum of care for patients, so we retrieved the following data from the database.

· Reviewed by a Cardiologist (NICOR | Reviewed by a Cardiologist)

· Performance of an angiogram (NICOR | Performance of an angiogram)

· Discharge on appropriate medications (NICOR | Discharge on appropriate medications)

· Timeliness of primary Percutaneous Coronary Intervention (PCI) by the hospital (NICOR | Timeliness of primary PCI by the hospital)

· Referral to cardiac rehabilitation (NICOR | Referral to cardiac rehabilitation)

After obtaining datasets from the MINAP database, data of specified variables was transferred to IBM SPSS Statistics for Windows, Version 28 (Released 2021; IBM Corp., Armonk, New York, United States), which was used to analyze the retrieved data. The data for different regions of the UK (England, Northern Ireland, and Wales) was separated from the original dataset. The Chi-square test was applied to find the association between other categorical variables. P-value <0.05 was considered statistically significant.

## Results

The data of 85574 ACS patients was retrieved. The majority (n=78158, 91.33%) of these patients were from England, while 3211 (3.76%) were from Northern Ireland and 4205 (4.91%) were from Wales. The patient’s data was retrieved from 197 healthcare settings in the United Kingdom, of which 175 healthcare settings were in England, 10 in Northern Ireland, and 12 in Wales.

**Figure 1:**
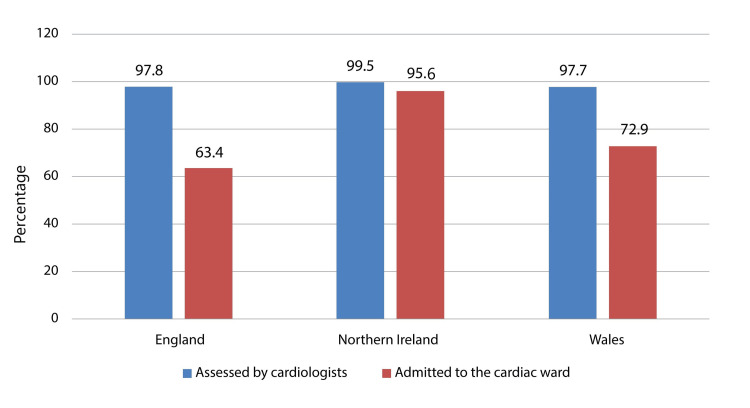
Acute coronary syndrome (ACS) patients assessed by cardiologists and admitted to cardiac wards in different regions of the United Kingdom In England, 97.8% of the eligible patients were assessed by cardiologists and 63.4% of the eligible patients were admitted to the cardiac ward. In Northern Ireland, 99.5% of the eligible patients were assessed by cardiologists and 95.6% of the eligible patients were admitted to the cardiac ward while in Wales 97.7% of the eligible patients were assessed by cardiologists and 72.9% of the eligible were admitted to the cardiac ward.

Of the total patients, 54768 were eligible to be assessed by a cardiologist; however, 53630 (97.9%) were assessed by cardiologists, and 35556 (64.9%) were subsequently admitted to the cardiac ward. In England, 49319 (97.8%) patients were assessed by cardiologists, and 31966 (63.4%) were admitted to the cardiac ward. In Northern Ireland, 1723 (99.5%) patients were assessed by cardiologists and 1660 (95.6%) were admitted to the cardiac ward, while in Wales, 2588 (97.7%) patients were assessed by cardiologists and 1930 (72.9%) were admitted to the cardiac ward. Though the proportion of patients admitted to cardiac wards was substantially less in England than in Northern Ireland and Wales, there was no significant difference between different regions of the United Kingdom and the percentage of patients assessed by cardiologists and patients admitted to cardiac wards (Figure 1).

**Figure 2:**
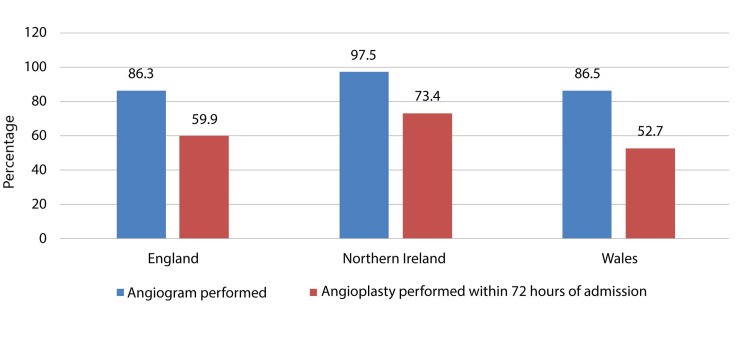
Acute coronary syndrome patients for whom angiogram and angioplasty were performed in different regions of the United Kingdom In England, an angiogram was performed for 86.3% of the eligible patients, while in Northern Ireland, an angiogram was performed for 97.5%. In Wales, it was performed for 86.5% of the eligible patients. In England, angioplasty within 72 hours of admission was performed for 59.9% of the eligible patients while in Northern Ireland angioplasty within 72 hours of admission was performed for 73.4% and in Wales, it was performed for 52.7% of the eligible patients.

A large number (n=47035) of patients were estimated to be eligible for an angiogram; however, an angiogram was performed for 87.15% (n=40995) of patients eligible for the angiogram. Of the total patients eligible for an angiogram, 43332 (92.1%) were from England, 1577 (3.35%) were from Northern Ireland, and 2396 (5.09%) were from Wales. In England, an angiogram was performed for 86.3% (n=37383) of the eligible patients. In contrast, in Northern Ireland, an angiogram was performed for 97.5% (n=1538); in Wales, it was performed for 86.5% (n=2074) of the eligible patients. Angioplasty within 72 hours of admission was required for most (n=26313) ACS patients. Nonetheless, angioplasty within 72 hours of admission was performed for 59.7% (n=15703) of the eligible patients. Of the total patients for whom angioplasty was required within 72 hours of admission, 24371 (92.6%) were from England, 823 (3.12%) were from Northern Ireland and 1119 (4.25%) were from Wales. In England, angioplasty within 72 hours of admission was performed for 59.9% (n=14509) of the eligible patients, while in Northern Ireland, angioplasty within 72 hours of admission was performed for 73.4% (n=604), and in Wales, it was performed for 52.7% (n=590) of the eligible patients. There was no significant difference between different regions of the United Kingdom and the percentage of patients for whom an angiogram was performed out of the eligible patients. However, there was a significant difference between different regions of the United Kingdom and the percentage of patients for whom angioplasty was performed within 72 hours of admission out of the eligible patients for angioplasty within 72 hours of admission (Figure 2).

Primary percutaneous coronary intervention (PCI) was performed for 23923 ACS patients, of which the door-to-balloon interval for 17590 (73.5%) patients was ≤60 minutes while the door-to-balloon interval for 3086 (12.9%) patients was ≤90 minutes. Of the total patients who were eligible for PCI, 21674 (90.6%) were from England, 989 (4.14%) were from Northern Ireland, and 1260 (5.27%) were from Wales. There was no significant difference between different regions of the United Kingdom and the percentage of patients who underwent PCI. Out of the total 85574 ACS patients, 65959 (77.08%) patients were discharged on appropriate medications (aldosterone antagonists in patients with echocardiographically proven poor left ventricular function, and a combination of angiotensin-converting enzyme inhibitors, angiotensin receptor blockers, aspirin, other antiplatelet agents, beta blockers and statins in all patients). In contrast, 19615 (22.92%) were transferred to another hospital or died there. A total of 75361 were eligible to be referred to cardiac rehabilitation settings. Nonetheless, 64518 (85.61%) of the eligible patients were referred to cardiac rehabilitation.

## Discussion

The research studies reported that recent advancements in the diagnosis and treatment of ACS resulted in better outcomes in ACS patients. There is consensus in the literature that the care ACS patients receive at each management stage defines the short- and long-term outcomes. Moreover, the clinical characteristics with which ACS patients present are partly responsible for the prognosis [25-27]. The current study reported clinical trajectories and the continuum of care for patients with ACS in the UK. The results showed that despite a developed healthcare system, disparities exist across different regions of the UK. It is pertinent to mention that most patients received the required care. However, disparities still exist, which might impact ACS's recognition, management, and outcomes.

Evidence suggests that substantial progress has been made in diagnosing and treating ACS, mainly in high-income countries [28,29]. However, the results of the current study showed that not all ACS patients eligible to be reviewed and assessed by cardiologists have accessed cardiologists for their check-ups. Though only a small proportion of eligible ACS patients were not evaluated by cardiologists, it signifies the need to enhance the accessibility, affordability, and timely provision of healthcare services for ACS patients. Similarly, the current study results showed that some proportions of eligible patients were not admitted to cardiac wards. This again highlights the need to strengthen the healthcare system in the UK further. Literature suggests that the “Health-for-all” concept can eliminate discrepancies in healthcare services, which means that equitable healthcare services should be provided irrespective of racial and socioeconomic differences [30,31].

Similarly, health insurance can minimize healthcare service disparities [32]. In the current study, we could not specifically explore why cardiologists could not review a certain number of patients and subsequently admit them to cardiac wards when they were eligible to be assessed by cardiologists and admitted to cardiac wards. Moreover, we could not explore the association of racial and socioeconomic disparities with the provision of care to ACS patients in the UK as it was beyond the objectives of the current study.

The results of the current study showed that a large proportion of ACS patients were eligible for the angiogram. However, an angiogram was performed for about 87% of the eligible patients. The majority of the patients received the needed care; however, a small proportion of ACS patients did not receive the required care. This again highlights the discrepancies in the present healthcare system because an angiogram is the main diagnostic option in ACS patients. Not performing this critical diagnostic tool in ACS patients may mislead in treating ACS patients [33,34]. Angiograms not only assist clinicians in the decision-making related to the care of ACS patients but also help to predict the prognosis of the condition [35,36]. Some evidence shows that a timely angiogram helps reduce morbidity and mortality associated with cardiac conditions, as an angiogram defines a care pathway for patients presenting with cardiac conditions [37-39].

Similarly, angioplasty within 72 hours of admission was required for most of the ACS patients. However, only about 60% of patients underwent angioplasty within 72 hours of admission. Because we used a secondary dataset, it was beyond this study's scope to further explore why angioplasty was not performed for about 40% of the eligible patients.

Nevertheless, the literature suggests that not performing angioplasty when required can lead to adverse outcomes in ACS patients [40,41]. The finding is of utmost significance because ACS's short- and long-term consequences directly depend on the management during in-patient hospitalization. Literature suggests that timely angioplasty when required can improve the prognosis of ACS patients and significantly reduce rates in ACS patients [42-44]. Again, we could not map the outcomes of these 40% of patients who were eligible for angioplasty but did not undergo angioplasty.

Evidence suggests that the effectiveness of PCI is affected by the door-to-balloon interval [45,46], and findings of the current study showed that the door-to-balloon interval for almost three-quarters (73.5%) of patients who underwent PCI was ≤60 minutes. Though findings showed a delay in PCI for about one-quarter of patients, shorter door-to-balloon intervals showed that the emergency response was quite good. It is pertinent to mention that for patients who need PCI, the door-to-balloon interval is considered a crucial time because the shorter door-to-balloon interval is associated with significantly reduced morbidity and mortality in ACS patients [47,48]. The results of the current study further showed that about 85% of the eligible patients were referred to cardiac rehabilitation. Cardiac rehabilitation is an important step to prepare ACS patients to return to their regular life routine. Cardiac rehabilitation helps improve endurance and enables ACS patients to cope with the challenges associated with ACS [49-51]. 

The current study thoroughly explored clinical trajectories and the continuum of care for patients with ACS in the UK. However, it has some limitations. For example, the current study did not explore the association of racial and socioeconomic disparities with providing care to ACS patients in the UK. Similarly, the recent study used secondary data primarily collected for clinical purposes. In addition, the present study focused only on variables accessible via online datasets. Therefore, there is a risk that we might have missed some critical variables that were not available in the online datasets.

Moreover, the current study did not assess the factors for which required care was not provided to the ACS patients in the UK. Similarly, we could not specify the outcomes in those patients who did not receive the necessary care. These limitations signify that the results of the current study need to be interpreted with caution.

## Conclusions

About 85000 patients were reported in the UK (England, Northern Ireland, Wales). Optimal care was provided to a majority of patients in the UK. However, some patients received sub-optimal care, highlighting the disparity in the healthcare system. A large proportion of ACS patients were reviewed and assessed by cardiologists. However, the number of patients admitted to cardiac wards out of the eligible patients was low. Similarly, an angiogram was performed on most patients eligible for an angiogram. However, angioplasty was not performed on more than one-third of ACS patients suitable for angioplasty. In addition, the balloon interval for three-quarters of the patients who underwent PCI was ≤ 60 minutes; however, the balloon interval for about one-quarter of the participants was >60 minutes. These findings show that despite a sustainable healthcare system, disparities exist in providing healthcare services to ACS patients in the UK. There is a need to explore further the factors that might be responsible for the sub-optimal care to the patients. Particularly, racial and socioeconomic disparities need to be explored in detail because these factors may be partly responsible for the discrepancies in the provision of healthcare services to ACS in the UK. Moreover, as there were limitations in the current study, high-quality, multicentre primary research studies need to be conducted to assess the clinical trajectories and the continuum of care for patients with ACS in the UK.
